# Baltimore College of Dental Surgery

**Published:** 1840-07

**Authors:** 


### BALTIMORE COLLEGE OF DENTAL SURGERY.

Our readers will perceive, by reference to our advertising page, that an institution has been established in Baltimore, under the above designation.

We heartily congratulate the dental profession upon this event—for event it is—in the progress of that science to which we have devoted ourselves, and in the advancement of which, every dentist worthy of the name feels deep interest.

There is no good reason for the inferior rank, that dental surgeons have been compelled to take, in comparison with professional men.—Why is the surgeon, who amputates a limb, or dresses an ulcer, more highly to be esteemed than he who confines his attention to the diseases of the mouth and dental arch ? Is the knowledge of the former acquired with more laborious industry ? Is his skill the result of more persevering research and careful experience ? Does the operations he is called upon to perform, require more judgment or greater nicety than those which devolve upon the practitioner of dentistry ? No man acquainted with the facts, in each case, will respond affirmatively to these inquiries. He who devotes his life to the alleviation of the sufferings of his fellows, is a *respectable* man ; and he who brings knowledge, carefully acquired, to aid in this object, is a *scientific* man —It matters not whether his efforts be directed to assuage a fever, or remove a pain,—to save life, or to render it comfortable—the object, the honor, the motive is the same,—and so it should be, in the regard of those who are profitted thereby.

Why then, we again ask, has the practice of dentistry held so low a place in public estimation ? The answer is ready,—because a large number of dentists, so called, are neither capable nor worthy. The public have rarely hitherto been able to distinguish between the man of science and the impudent adventurer. Dentistry has been one of those three cities of refuge to the ignorant and unfortunate ; and very often when the choice was presented to the mind of an individual scheming for existence, between an office under government, enlisting in the army, or practising dentistry, the latter has been preferred as more accessible than the first, and less laborious than the second. All know the result of this evil. Men, who are conscious of deserving nothing, are ready to labor for half price. Honesty must die in such competition, or survive like an humble flower overshadowed by rank weeds.

Knowing these things to be so, we cannot but rejoice in the establishment of an institution for the education of dentists. We are glad too, that the college has been arranged upon a liberal scale. A dental surgeon ought to be sufficiently well informed to make him a companion for the best educated physicians and surgeons in the land. He ought no longer to be satisfied with a secondary station ; and should the present effort be sustained, and we doubt not but it will be, he will no longer be compelled to be contented with it.

Two-thirds of the board of visitors, are medical men,—this is as it should be. In the noble pursuit of science, there is no room for petty jealousy. It has its seat only in contracted minds ;—there let it remain, buried where it can injure none, save the heart that harbours it.

